# In Situ Tumor Vaccination Using Lipid Nanoparticles to Deliver Interferon-β mRNA Cargo

**DOI:** 10.3390/vaccines13020178

**Published:** 2025-02-13

**Authors:** Kenji Kimura, Aidan Aicher, Emma Niemeyer, Phurin Areesawangkit, Caitlin Tilsed, Karen P. Fong, Tyler E. Papp, Steven M. Albelda, Hamideh Parhiz, Jarrod D. Predina

**Affiliations:** 1 Division of Pulmonary Allergy and Critical Care, Department of Medicine, Perelman School of Medicine, University of Pennsylvania, Philadelphia, PA 19104, USAkpyi@pennmedicine.upenn.edu (K.P.F.);; 2 Division of Thoracic Surgery, Department of Surgery, Perelman School of Medicine, University of Pennsylvania, Philadelphia, PA 19104, USA; 3 Department of Systems Pharmacology and Translational Therapeutics, Perelman School of Medicine, University of Pennsylvania, Philadelphia, PA 19104, USA; typapp@pennmedicine.upenn.edu; 4 Penn Institute for RNA Innovation, University of Pennsylvania, Philadelphia, PA 19104, USA

**Keywords:** in situ vaccination, intratumoral immunotherapy, interferon, lipid nanoparticles

## Abstract

*Background:* In situ cancer vaccination is a therapeutic approach that involves stimulating the immune system in order to generate a polyclonal, anti-tumor response against an array of tumor neoantigens. Traditionally, in situ vaccination approaches have utilized adenoviral vectors to deliver immune-stimulating genes directly to the tumor microenvironment. Lipid nanoparticle (LNP)-mediated delivery methods offer several advantages over adenoviral delivery approaches, including increased safety, repeated administration potential, and enhanced tumor microenvironment activation. *Methods:* To explore in situ vaccination using LNPs, we evaluated LNP-mediated delivery of a reporter gene, mCherry, and an immune-stimulating gene, IFNβ, in several in vitro and in vivo models of lung cancer. *Results:* In vitro experiments demonstrated successful transfection of murine cancer cell lines with LNPs carrying both mCherry and IFN-β mRNA, resulting in high expression levels and IFNβ production. In vivo studies using LLC.ova flank tumors showed that intratumoral injection of IFNβ-mRNA LNPs led to significant IFNβ production within the tumor microenvironment, with minimal systemic exposure. Therapeutic efficacy was evaluated by injecting established LLC.ova flank tumors with IFNβ-mRNA LNPs bi-weekly for two weeks. Treated tumors showed significant growth inhibition compared to controls. Flow cytometric analysis of tumor-infiltrating leukocytes revealed that tumors injected with IFNβ-mRNA LNPs were associated with an increased CD8:CD4 T-cell ratio among lymphocytes, more CD69-expressing CD8 T-cells, and an increased presence of M1 macrophages. Efficacy and an abscopal effect were confirmed in a squamous cell carcinoma model, MOC1. No toxicity was observed. *Conclusions:* These findings show that intratumoral LNP delivery of immune-stimulating mRNA transcripts, such as IFNβ, can effectively stimulate local anti-tumor immune responses and warrants further investigation as a potential immunotherapeutic approach for cancer.

## 1. Introduction

The effects of type 1 interferons (namely, interferon-α and interferon-β) in the tumor microenvironment (TME) are extensive [1,2,3,4]. Direct actions on cancer cells within the TME include decreased proliferation, induction of cell death with the release of neoantigens and danger signals, upregulation of MHC Class 1 molecules and tumor antigen expression, and upregulation of PD-L1 and IDO1. The TME is also significantly altered through a variety of mechanisms including inhibition of tumor neovascularization, widespread activation of innate immune cells (including NK cells, dendritic cells, and macrophages), reprogramming of myeloid-derived suppressor cells, and a reduction in T-reg infiltration. These effects (with the exception of increased PD-L1 and IDO1) result in enhanced anti-tumor-directed CD8 and CD4 T-cells and reductions in tumor growth.

Despite these strong anti-tumor effects, the clinical use of type 1 interferons has been limited by toxicity, lack of tumor targeting, and a short half-life [1,2,3,4]. One approach to address these issues has been local tumoral delivery using gene therapy. Direct instillation of a replication-deficient adenovirus encoding IFNα into patients with minimally invasive bladder cancer has shown anti-tumor efficacy [5] and was recently approved for use by the FDA. Further, our group has similarly used replication-deficient adenoviral vectors to deliver both IFNα and IFNβ to patients with thoracic malignancies [6,7,8,9,10].

For this strategy to be more broadly applicable (i.e., effective in metastatic tumors), an abscopal effect is required. That is, the local injection needs to serve as an “in situ vaccination” that induces the production of a systemic anti-tumor immune response [11,12]. Our group, as well as others, have shown that systemic immune effects can indeed be induced with replication-deficient adenoviral vectors encoding interferon-α (IFNα) and interferon-β (IFNβ) in preclinical models and clinical trials of intrapleural injection in patients with malignant mesothelioma and metastatic pleural effusions [6,7,8,10,13,14].

Although gene therapy utilizing adenoviral vectors to deliver immune-stimulating genes has provided an important “proof of principal”, there are disadvantages associated with adenoviral vectors. For example, clinical-grade products are expensive to produce and store. Manufacturing is also challenging, particularly when viruses are used to deliver products (like interferon) that, paradoxically, limit viral production. Adenoviruses also induce strong anti-viral immune responses that can potentially overshadow anti-tumor immune responses, produce systemic side effects, and result in a rapid production of high titers of neutralizing antibodies (Nab). Nabs can further limit the efficacy of repeat injections of the vector [15]. Finally, Type 5 adenoviruses commonly used clinically are not able to infect leukocytes, thus preventing direct effects on white blood cells and limiting the number of cells that might be able to produce a specific cargo.

Given the recent advances in mRNA delivery using lipid nanoparticles [16], we hypothesized that intratumoral (IT) delivery of a type 1 interferon (IFNβ) mRNA using lipid nanoparticles (LNPs) would display similar efficacy as adenoviral constructs but have advantages over adenoviral delivery because of (1) increased safety, (2) the ability to administer repeated injections, (3) increased tumor microenvironment activation due to the intrinsic adjuvant properties of the LNPs, (4) the ability to infect dendritic cells (DCs), macrophages, and other leukocytes, and (5) the potential to multiplex cargos.

Accordingly, in this report, we explore the efficacy of intratumoral delivery of LNPs (using the SM102 ionizable lipid) containing optimized mRNA for (1) a reporter gene, mCherry, and (2) murine interferon-β.

## 2. Materials and Methods

### 2.1. The Cell Lines and Tumor Models

The following murine cancer cell lines were used: (1) a lung cancer cell line (LLC) expressing chicken ovalbumin (Ova) provided by Richard G. Vile (Molecular Medicine, Mayo Clinic), (2) TC1 (derived from mouse lung epithelial cells immortalized with HPV-16 E6 and E7 and transformed with the c-Ha-ras oncogene as previously noted [13]) purchased from the ATTC, and (3) oral carcinoma (MOC1) cells obtained from Kerafast (Boston, MA, USA). Cells were cultured in Roswell Park Memorial Institute medium, 10% (vol/vol) FBS, 2 mmol/L glutamine, and 1% (vol/vol) penicillin/streptomycin and were tested regularly and maintained negative for Mycoplasma spp. For tumor studies, 1 *×* 10^6^ tumor cells suspended in 100 μL PBS were injected subcutaneously. Tumor volume was calculated using the equation (3.14 × length of longest dimension × length of shortest dimension/2)/6 [13].

### 2.2. Animals

Female C57BL/6 mice (B6, Thy1.2) were purchased from Charles River Laboratories. Mice were used for experiments at age 8 weeks or older. Animals were maintained in pathogen-free conditions, recognized principles of laboratory animal care were followed, and the Animal Use Committees of the University of Pennsylvania approved all protocols.

### 2.3. Generation of LNPs to Deliver mCherry or IFNβ mRNA

The DNA coding sequence for mCherry fluorescent protein was obtained from SnapGene, v8.0 (www.snapgene.com/resources, accessed on 30 December 2024) software and then codon optimized. The murine IFNb gene was obtained from the NCBI reference sequence X14455.1 and then codon-optimized. The mRNA sequences were then individually cloned into an in vitro transcribed mRNA (IVT-mRNA) production template plasmid carrying a T7 promoter, 5′ and 3′ UTR elements, a Kozak consensus sequence, and a 101 poly(A) tail. DNA synthesis, cloning, and industrial-grade endotoxin-free plasmid preparation were provided by GenScript. IVT-mRNA was synthesized using linearized IVT template plasmid and the MEGAScript T7 kit (Thermo Fisher) then formulated with nucleoside-modified m1Ψ-5′-triphosphate (TriLink, San Diego, CA, USA, N-1081) alternatively to UTP. Next, 5′ capping of the IVT-mRNA was performed co-transcriptionally using the trinucleotide cap1 analog, CleanCap*^®^* Reagent AG (3′ OMe) (TriLink, N-7413). Single-stranded IVT-mRNA was purified through cellulose purification, as was previously described [17]. The mRNA was analyzed via agarose gel electrophoresis and stored at −20 °C.

### 2.4. LNP Encapsulation

To encapsulate cellulose-purified m1Ψ-containing mCherry or IFNβ mRNAs in LNPs, we used a self-assembly process that has been previously described [18]. Briefly, briefly an ethanolic lipid mixture of phosphatidylcholine, cholesterol, SM-102 ionizable cationic lipid, and polyethylene glycolipid was rapidly mixed with an aqueous solution containing the mRNA at acidic pH with a NanoAssemblr™ Ignite™ (Cytiva, Marlborough, MA, USA). Dynamic light scattering using a Zetasizer Nano ZS (Malvern Instruments, Malvern, UK) and a Quant-iT Ribogreen assay (Thermo Fisher) were used to characterize the RNA-loaded particles. The mean hydrodynamic diameter of these LNP-mRNAs was approximately 80 nm with a polydispersity index of 0.02–0.06 and an encapsulation efficiency of ~95%. mRNA content was calculated by performing a modified Quant-iT RiboGreen RNA assay (Thermo Fisher). LNPs were stored in −80 °C.

### 2.5. In Vitro Delivery of mCherry or INFβ mRNA Using LNPs

Using a 96-well plate, cells were plated at 1.5 × 10^4^ cells/well and allowed to rest for 6 h in 200 μL of media. Cells were then washed with cold PBS and were co-cultured with media spiked with LNPs at varying concentrations of LNPs for 24 h. Unless otherwise noted, concentrations are described as μg of mRNA per 1 × 10^6^ cells. To determine the expression of mCherry protein, cells were lifted from plates with trypsin and then stained for flow cytometric analysis. To determine the expression of IFNβ, supernatants were collected and analyzed using an IFNβ ELISA (R&D Systems, Minneapolis, MN, USA, Catalog # MIF00).

### 2.6. 3-(4,5-Dimethylthiazol-2-yl)-5-(3-carboxymethoxyphenyl)-2-(4-sulfophenyl)-2H-tetra-zolium (MTS) Assay

To determine cell viability, 1.0 × 10^5^ cells were plated into wells of a flat-bottomed 96-well plate and cultured in media overnight. The next morning, the media was removed and replaced with 0.2 mL of media containing LNPs with varying concentrations of mRNA per million cells. The plates were incubated at 37 °C for 24 h. Next, 20 µL of the MTS labeling reagent (CellTiter 96 AQueous One Solution Cell Proliferation Assay, Promega, Madison, WI, USA) was added to each well. Plates were incubated at 37 °C for three hours. The spectrophotometric absorbance was assessed with a VarioScan Flash microplate reader (Thermo Fisher Scientific, Waltham, MA, USA) at 480 nm, with a reference of 630 nm.

### 2.7. Intratumoral Delivery of LNPs

Animals bearing flank tumors were treated with intratumoral (IT) injections of LNPs. In these studies, the dose was defined by the amount of mRNA delivered (10 μg, unless otherwise indicated). LNPs were suspended in 30–50 μL of cold PBS and injected using a 27 G insulin syringe.

### 2.8. Flow Cytometric Analysis of Tumors

Samples containing single-cell suspensions were obtained by removing tumors and livers from euthanized mice and mincing these into fine pieces in a digestion buffer containing 0.1 mg/mL DNase I and 2.0 mg/mL collagenase type IV (Sigma, St. Louis, MO, USA). Samples were incubated in a digestion buffer at 37 °C for 30 min, filtered through a 70-μm filter, and washed twice with R10. After obtaining a single-cell suspension, anti-mouse CD16/CD32 antibodies (BD Biosciences, Franklin Lakes, NJ, USA) were used to block Fc receptors. After washing with PBS plus 2% FBS (staining buffer), cells were incubated for 30 min at 4 °C with appropriate antibodies (anti-CD45, CD4, CD8, CD19, CD11b, CD11c, Ly6G, Ly6C, F4/80, CD90, and CD31). Samples were then washed and resuspended in staining buffer or fixed in 2% paraformaldehyde. Flow cytometry was conducted using a Becton Dickinson FACS Calibur flow cytometer and analyzed using FlowJo software, v10.1.

### 2.9. Statistical Analyses

For experiments comparing differences among two groups, an unpaired Student’s t-test was utilized. For those studies comparing more than two groups, ANOVA with appropriate post hoc testing was utilized. All in vivo experiments utilized 5 mice per group, unless otherwise noted. Differences were considered significant when *p* < 0.05. Data are presented as mean ± SEM unless otherwise noted.

## 3. Results

### 3.1. Delivery of Marker Genes to Tumor Cells In Vitro Using Untargeted SM102-Based LNPs

**Figure 1:**
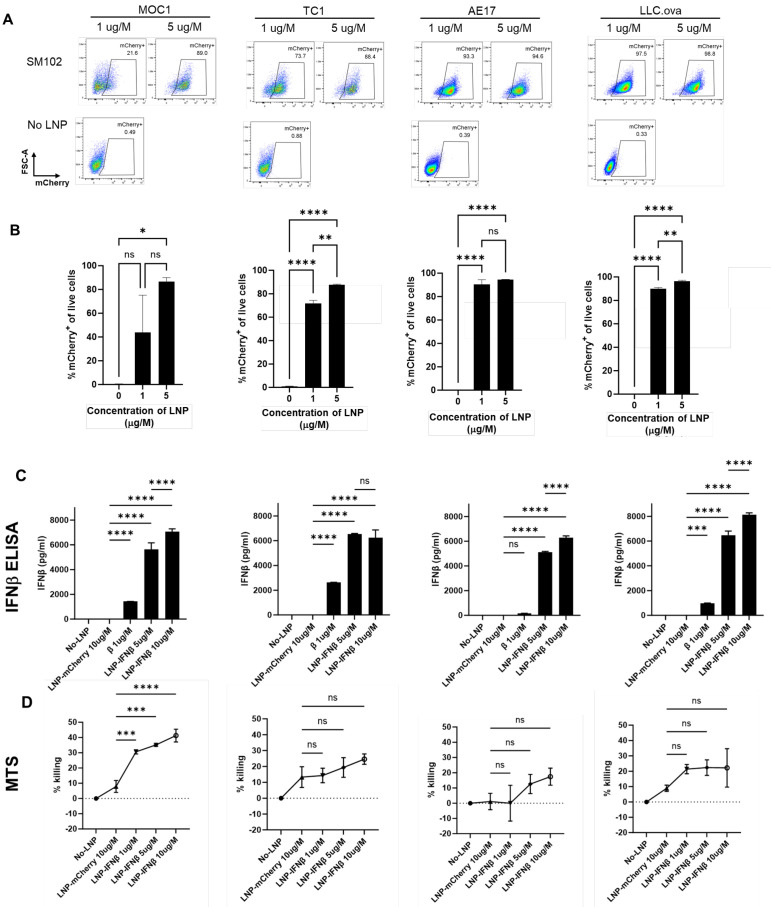
LNPs successfully deliver mRNA transcripts to cancer cells in vitro. Four murine cancer lines were cultured with media containing two doses of mCherry-LNPs for 24 h. Cells were then harvested and evaluated using flow cytometry; representative flow cytometry tracings are provided (**A**) and summary statistics are displayed (**B**). The same cancer lines were cultured with media containing 3 doses of IFNβ-LNPs for 24 h. Supernatants were collected and analyzed by IFNβ ELISA (**C**). The toxicity of IFNβ-LNP was evaluated by assessing viability using a standard MTS Assay (**D**). μg/M (μg of LNP per Million cells), pg/mL (pg of IFN per mL of supernatant), ns-not significant, * *p* < 0.05, ** *p* < 0.01, *** *p* < 0.001, **** *p* < 0.0001.

Untargeted LNPs generated using the ionizable lipid, SM102, encapsulating mRNA encoding for the reporter gene, mCherry (mCherry-LNP), were added at two doses to a series of murine cancer cell lines (LLC, MOC1, TC1, and AE17). After 24 h of co-culture, the harvested tumor cells were found to express mCherry at high levels upon flow cytometric analysis (Figure 1A,B), showing that LNPs carrying mCherry mRNA cargo are able to efficiently transfect murine cancer cells in vitro.

### 3.2. In Vitro Production of IFNβ Induced by IFNβ-mRNA LNPs

We next evaluated the ability of SM102 LNPs to deliver mRNA-encoding murine IFNβ (IFNβ-LNP) in vitro. LNPs carrying mRNA for mIFNβ were added to murine cancer cell lines at varying concentrations, and supernatants were harvested after 24 h, and analyzed by ELISA. Each cell line produced high levels of IFNβ in a dose-responsive fashion (Figure 1C). Since type I interferons are known to induce cell death in some tumor cell lines [2], we evaluated the cytotoxicity of LNP-IFNβ in our cell lines after 24 h of co-culture using an MTS assay. The addition of LNPs carrying IFNβ mRNA led to varying degrees of cell killing ranging from ~20% (LLC.ova, TC1, AE17) to 40% in MOC1 cells (Figure 1D). Together, these data confirmed that LNPs carrying INFβ mRNA were capable of in vitro delivery of mRNA, which resulted in translation and the secretion of the INFβ protein. Further, the expression of INFβ resulted in modest amounts of cellular killing.

### 3.3. In Vivo Expression of Transgenes After Intratumoral LNP Injection

After establishing that SM102-based LNPs were capable of mRNA transfection in vitro, we focused on initial in vivo efficacy using the LLC.ova cell line. Of note, LLC.ova is a relatively “hot” tumor, with approximately 20% of the tumor composed of leukocytes. Of these leukocytes, the majority are myeloid cells (macrophages and neutrophils) with tumor-infiltrating T-cells accounting for approximately 6% (Supplemental Figure S1).

**Figure 2:**
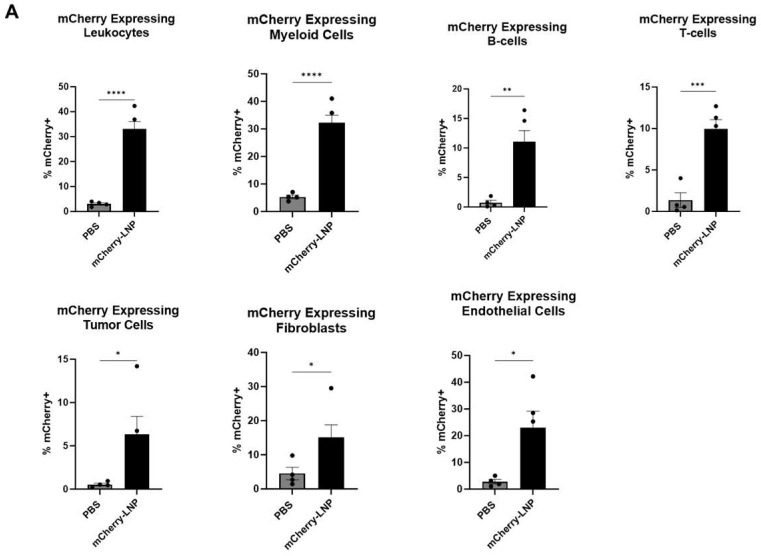

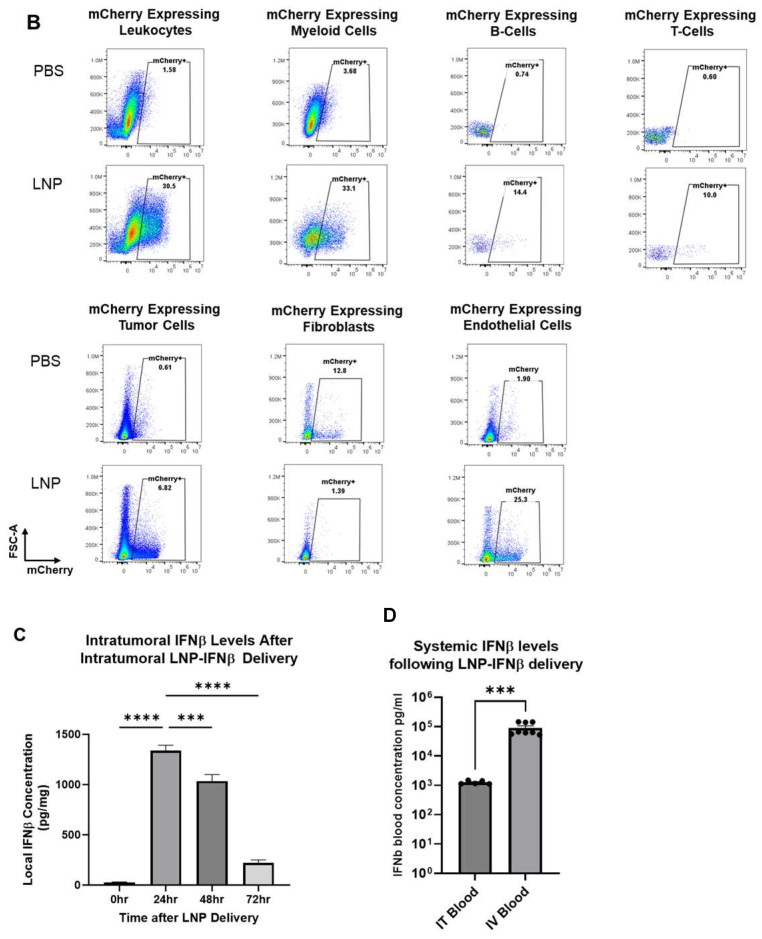
LNPs effectively deliver transcripts to flank tumors. Immunocompetent mice bearing LLC.ova flank tumors were injected intratumorally with 10 μg mCherry-LNPs. After 24 h, tumors were analyzed using flow cytometry to determine which cellular populations had been successfully transfected with LNPs (**A**). Representative flow cytometry tracings are provided (**B**). Mice bearing LLC.ova tumors were injected with 10 μg IFNβ-LNPs and tumors were homogenized at designated time points after injection. IFNβ levels were assessed using ELISA and the results are normalized by tumor weight (mg) (**C**). Systemic INFβ levels were assessed using ELISA 24 h after intratumoral and intravenous injection of PBS or 10 μg of IFNβ-LNPs (**D**). * IT-Intratumoral, IV-intravenous, pg/mg (pg of IFN per mg of tumor), pg/mL (pg of IFN per mL of blood), ns-not significant, * *p* < 0.05, ** *p* < 0.01, *** *p* < 0.001, **** *p* < 0.0001 μg/M.

Either 30 μL of PBS or 10 μg of mCherry-LNPs (in 30 μL) were injected intratumorally (IT) into established LLC.ova tumors that ranged from 100 to 150 mm^3^ in size. After 24 h, the tumors were harvested, digested, and subjected to flow cytometry analysis (Figure 2A,B). Approximately 33% of leukocytes (CD45+ cells) expressed mCherry, with myeloid (CD11b+) cells demonstrating the highest levels of expression (32%) among leukocyte sub-populations. We noted that 22% of endothelial (CD31+) cells expressed mCherry, and 17% of fibroblasts (CD90+ cells) expressed mCherry. Only about 6% of tumor (CD45-/CD31-/CD90-) cells expressed mCherry.

To confirm that SM102 IFNβ-LNPs could produce IFNβ protein in vivo, we injected similarly sized LLC.ova tumors with 30 μL of PBS or SM102 LNPs encapsulating 10 μg of IFNβ mRNA. Tumors were harvested 24, 48, and 72 h after LNP injection, minced, and placed in 2 mL of ice-cold PBS. Supernatants were collected and then analyzed using an IFNβ ELISA. High levels of IFNβ were detected at 24 h (1339 pg/mg tumor) and 48 h (1034 pg/mg), with detectable (221 pg/mg) but lower levels at 72 h (Figure 2C). We also compared the expression IFNβ in the blood after IT and IV injection of the LNPs. IFNβ was not detectable in the blood of tumor-bearing mice injected IV or IT with PBS control. IV injection of the LNPs led to very high systemic levels of IFNβ (10^5^ pg/mL), likely because of uptake and protein production in the liver. Although IFNβ was detected in blood after IT LNP injection, levels were ~100-fold lower (10^3^ pg/mL) than after IV injection (*p* < 0.001) (Figure 2D).

Taken together, these results demonstrate that LNPs are capable of transfecting LLC.ova tumor cells and that the mRNAs lead to successful protein translation in vivo. Additionally, IT tumor injections are associated with much lower systemic IFNβ levels than after IV injection.

### 3.4. Intraumoral Delivery of IFNβ-LNP Inhibits Tumor Growth in LLC.ova Flank Tumors

**Figure 3:**
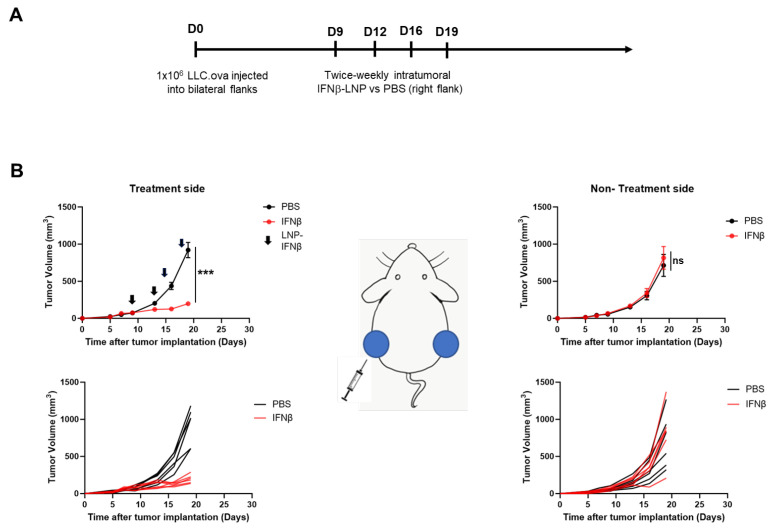
Intratumoral delivery of IFNβ-LNPs inhibits tumor growth in LLC.ova flank tumors. Mice bearing bilateral LLC.oval flank tumors were randomized to biweekly, unilateral injections of PBS or IFNβ-LNPs as depicted (**A**). Growth of flank tumors on the side of the injected tumors (**B**, left panels) and non-injected side (**B**, right panels). Of note, upper growth plots in panel (**B**) represent the mean (SEM); lower growth plots represent individual tumor growth. PBS-tumors injected with PBS, IFNβ—tumors injected with LNP-IFNβ, *** *p* < 0.001, arrow notes the time of injection.

Given these data, we evaluated the impact of intra-tumoral IFNβ-LNPs on flank tumor growth. Bilateral LLC.ova tumors were generated by delivering 1 × 10^6^ cells subcutaneously onto the right and left flanks of mice. After flank tumors were established and measured ~100 mm^3^ in volume, PBS or LNPs encoding for IFNβ (10 μg of mRNA) were delivered intratumorally to the right tumor. LNPs were delivered bi-weekly for 2 weeks (Figure 3A). In mice receiving IFNβ-LNPs, injected tumors were noted to be significantly smaller compared to those in mice receiving intra-tumoral PBS injections (176 mm^3^ versus 956 mm^3^; *p* < 0.001) after two weeks (Figure 3B, left panels). We did not, however, observe reductions in tumor volume on the side contralateral to intra-tumoral injections (abscopal effects) (Figure 3B, right panels). After the cessation of IFNβ-LNP delivery, tumors resumed normal growth kinetics. During treatment, animals maintained body weights and displayed normal behavior, thus suggesting minimal toxicity.

**Figure 4:**
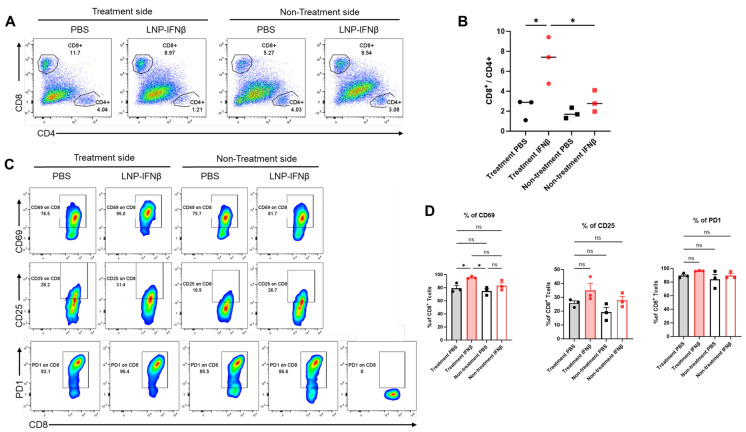
Intratumoral IFNβ-LNPs are associated with quantitative and qualitative changes in tumor-infiltrating CD8 lymphocytes. Within the TME, the percentage of CD8 and CD4 T-cells of all leukocytes was evaluated using flow cytometry. Sample tracings from mice injected with IFNβ-LNPs or PBS, stratified by injected side versus non-injected side, are provided (**A**). Graph demonstrating the ratio of CD8:CD4 T cells within TME (**B**). Infiltrating CD8 T-cells were evaluated for several activation markers and sample flow cytometry tracings (CD69, CD25, and PD1) (**C**), and summary statistics are provided (**D**). ns-not significant, * *p* < 0.05.

To determine the local immune responses driving differential tumor sizes, we repeated the above experiment and this time sacrificed mice 3 days after the second intratumoral injection of LNPs. We harvested both injected and non-injected flank tumors and then analyzed tumor digests using flow cytometry. At this time point, the percentage of lymphocytes within those tumors undergoing injection with IFNβ-LNPs was similar to that in control-treated tumors (~25% of the CD45+ cells); however, the percentage of CD8 T-cells of the lymphocytes vs. CD4 T-cells of lymphocytes was significantly higher in the INFβ treated tumors than PBS-treated tumors, resulting in a significantly higher CD8/CD4 T cell ratio (Figure 4A,B). This trend, however, was not observed in the tumors contralateral to LNP or PBS injections (Figure 4A,B).

We also found that CD8 T-cells harvested from tumors injected with IFNβ-LNPs displayed significantly higher levels of the activation marker, CD69 (Figure 4C,D), a change that was not seen in those tumors on the contralateral flank which did not receive intra-tumoral LNPs. There was a trend toward increased CD25 expression on the IFNβ-LNP injected tumors, but no difference in the expression of PD1 (Figure 4C,D).

**Figure 5:**
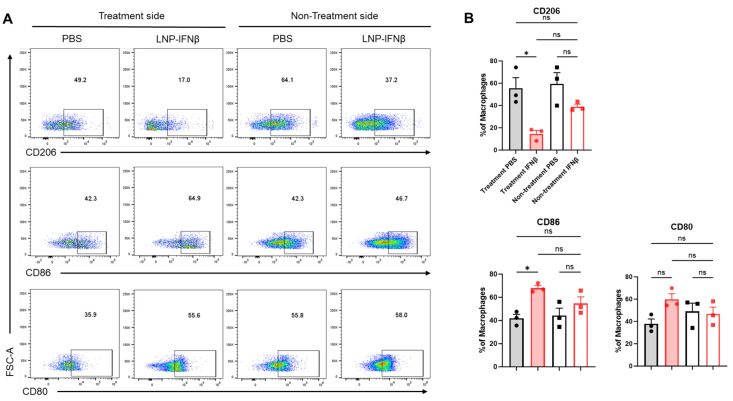
Intratumoral IFNβ-LNPs are associated with macrophages exhibiting an M1 phenotype. Within the TME, the F4/80+ macrophages were evaluated for several surface markers including CD206, CD86, and CD80. Representative tracings from mice injected with IFNβ-LNPs or PBS, stratified by injected side versus non-injected side, are provided (**A**). Graphs demonstrate the percentage of macrophages exhibiting CD206, CD86, or CD80 expression (**B**). ns—not significant, * *p* < 0.05.

In addition to differences in tumor-infiltrating lymphocytes, we observed phenotypic changes within the F480+ tumor-associated macrophage (TAM) population. Among the tumors injected with IFNβ-LNPs, TAMs displayed lower levels of the immunosuppressive M2 macrophage marker, CD206, along with increases in the anti-tumor M1 macrophage markers, CD80 and CD86 (Figure 5). These trends were not observed within those tumors contralateral to injections.

These results suggest that the intra-tumoral injection of LNPs carrying INFβ mRNA is associated with local lymphocytic and myeloid changes in the tumor microenvironment, favoring anti-tumor responses. Similar phenotypic changes were not observed distantly within contralateral tumors in the LLC.ova model, which may explain the lack of an abscopal effect in the LLC.ova model.

### 3.5. Effect of IFNβ-LNPs on a Second Tumor

**Figure 6:**
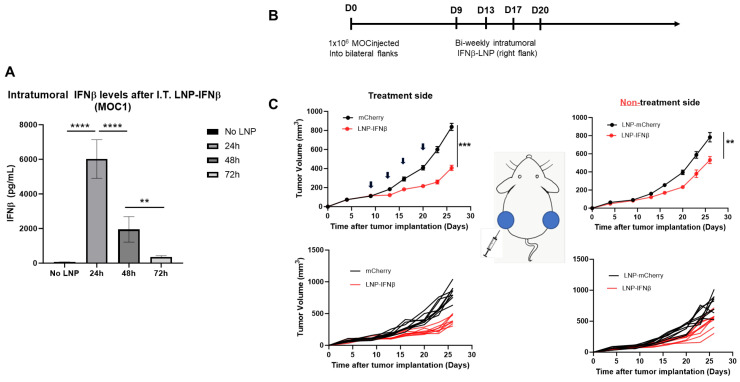
IFNβ-LNPs transfect MOC1 tumor cells in vivo and delay the growth of flank tumors. Mice bearing unilateral MOC1 flank tumors were injected with PBS or IFNβ-LNPs; tumors were then homogenized at several time points after injection. IFNβ levels were assessed by ELISA and results are normalized by tumor weight (mg) (**A**). To assess efficacy in vivo, mice bearing bilateral MOC1 flank tumors were randomized to biweekly, unilateral injections of PBS or IFNβ-LNPs as depicted (**B**). Growth of flank tumors on the side of the injected tumors (**C**, left panels) and non-injected side (**C**, right panels). Of note, upper growth plots in panel (**C**) represent the mean (SEM); lower growth plots represent individual tumor growth. ns-not significant, ** *p* < 0.01, *** *p* < 0.001, **** *p* < 0.0001.

Given the encouraging results observed in the LLC.ova model, we sought to examine the effects of intratumoral INFβ-LNPs in a second tumor line. For these studies, we utilized, MOC1, a murine model of squamous cell head and neck carcinoma. In contrast to LLC-ova, in our hands, MOC is a relatively “cold” tumor with low numbers of infiltrating leukocytes and T cells (Supplemental Figure S1). Similar to LLC.ova flank tumors, we first confirmed that IFNβ-LNPs deliver mRNA to MOC1 tumors in vivo. Established MOC1 tumors were injected with 30 μL of PBS or SM102 LNPs encapsulating 10 μg of IFNβ mRNA, harvested, and analyzed for the presence of IFNβ. Similar to observations in the LLC.ova flank tumors, high levels of IFNβ were detected at 24 h (5648 pg/mg tumor) and 48 h (1643 pg/mg), with detectable (346 pg/mg) but lower levels at 72 h (Figure 6A).

To evaluate the efficacy of INFβ-LNPs in MOC1 tumors, we again established bilateral flank tumors in C57/B6 mice. Once tumors reached approximately 100 mm^3^ in volume, IFNβ LNPs or mCherry LNPs (10 μg of mRNA) were delivered intratumorally to the right flank tumor. LNPs were delivered bi-weekly for 2 weeks (Figure 6B). In the injected tumors, we appreciated significant decreases in tumor volume after therapy (846 mm^3^ versus 364 mm^3^; *p* < 0.001) (Figure 6C, left panels). However, unlike the LLC.ova flank tumor models, we did observe reductions in tumor volumes on the side contralateral to intra-tumoral injections (790 mm^3^ versus 550 mm^3^; *p* < 0.01), representing an abscopal effect (Figure 6C, right panels).

## 4. Discussion

In this study, we establish that intra-tumoral (IT) injection of SM102-containing lipid nanoparticles encapsulating mRNA, similar to those used in the COVID-19 vaccines, are able to transfect tumor cells within murine tumor models. Marker proteins were expressed in cancer cells and other cells within the tumor microenvironment, especially in B cells and myeloid cells. IT injection of LNP’s containing the interferon-β mRNA resulted in protein translation and local secretion of interferon at high levels for up to 3 days within the tumor microenvironment. Interferon was observed at lower levels systemically. mRNA-induced IFNβ resulted in the activation of CD8 T-cells and TAMs and marked inhibition of tumor growth. This effect was not seen with control LNPs expressing mCherry. In addition, abscopal effects were noted in one model, as some decreases in tumor growth were observed in non-injected tumors. No adverse effects were noted and multiple doses were well tolerated.

These results compare favorably with our previous studies using an adenoviral vector expressing the same interferon transgene [7,8,9,10,14,17,18,19]. This is encouraging, as Ad.IFN has been used successfully in a number of clinical trials in patients with bladder carcinoma [20,21] and malignant pleural effusions [8,9]. However, one important advantage of LNP-based delivery we noted in the animal models was that the LNP’s were able to transduce myeloid cells in addition to tumor cells, whereas Ad vectors transfect epithelial cells with little to no uptake in leukocytes [22]. Since myeloid cells are very efficient producers of cytokines, their transduction likely augments changes in the TME, which include reprogramming of leukocytes.

Reports of IT delivery of interferon mRNA via LNP vectors are limited; however, there are several reports involving the intra-tumoral delivery of various other immune-stimulating transcripts using LNPs. Given its potent immune-stimulating activity but strong systemic side effects, intratumoral interleukin-12 (IL-12) mRNA alone or in combinations has been studied the most. For example, an intravenously injected LNP encasing IL12 mRNA (provided by Moderna, Cambridge, MA, USA) was used to treat a transgenic hepatocellular cancer model, and anti-tumor effects were observed [23]. Hewitt and scientists from Moderna reported that a single IT dose of LNPs encapsulating mouse mIL12 mRNA induced IFNγ and CD8 T-cell–dependent tumor regression in multiple syngeneic mouse models and exhibited abscopal effects [24]. Li et al. have described an LNP encapsulating self-replicating RNAs (replicons) that encoded an IL-12 cytokine fusion protein that was able to successfully combine robust ICD, inflammatory cytokine expression, and innate immune stimulation following IT injection and exert strong anti-tumor effects [25]. Additionally, Liu et al. [26] showed that IT delivery of IL-12 and IL-27 mRNAs had strong anti-tumor effects and induced robust infiltration of immune effector cells, including IFN-γ and TNF-α producing NK and CD8+ T cells into tumors. IT injection of the Moderna IL12 mRNA LNP (called MEDI1191) in combination with intravenous durvalumab entered a clinical trial in 2019 (NCT03946800).

In addition to the delivery of IL12 mRNA, others have explored multiplexed LNPs to deliver other immune-stimulating mRNA transcripts directly into the tumor. For example, Van der Jeught and colleagues described IT delivery of an mRNA encoding a fusion protein of interferon-β and the ectodomain of TGFβ receptor II and showed some anti-tumor activity that was enhanced by anti-PD1 antibodies [27]. Hotz et al. investigated the intra-tumoral delivery of saline-formulated mRNA encoding a four-component injection mixture of cytokine mRNAs (the components being active IL-15 (a fusion protein of IL-15 and the sushi domain of IL-15Ra), single-chain IL-12 (a fusion protein of p35 and p40) a granulocyte–macrophage colony-stimulating factor (GM-CSF), and interferon-alpha 2b) and showed strong anti-tumor activity [28]. Less efficacy was seen with individual components, including IFNα. Yang et al. described preclinical studies using the same four-component mixture but delivered as IT injections of circular mRNA. Although this mixture had anti-tumor activity, the individual components were not tested [29]. In another combination approach (Triplet LNP), LNPs delivering an mRNA mixture of encoding cytokines IL-2 and IL-7, as well as the immunostimulatory molecular 41BB ligand, resulted in potent CD8 T-cell activation and the development of long-term immunologic memory [30]. Additionally, Hewitt and colleagues have used LNPs to intra-tumorally deliver IL36γ, IL23, and Ox40L mRNAs [31]. This multiplexed construct induced downstream cytokine and chemokine expression and anti-tumor effects that were enhanced by immune checkpoint blockade. The mixture is being tested in a clinical trial alone and in combination with durvalumab (NCT03739931).

The significant anti-tumor effects observed with LNP-IFNβ noted in our study thus serve as an important proof of principle, and provide preliminary data that supports future studies investigating intra-tumoral delivery of IFNβ mRNA using LNPs. The lower systemic toxicity of IFNβ compared with IL-12 could represent an important advantage. In addition, we believe that future studies should focus on augmenting the observed anti-tumor effects. One such opportunity could involve multiplexing mRNA transcript formulations that have been described previously. Additionally, given the deleterious effect of interferons on immunotherapy by upregulation of PDL-1 on tumor cells and macrophages [32], another relatively obvious strategy would be to co-administer antibodies that block PD1 or PDL-1. Similarly, IDO1 is also upregulated by type 1 interferons, suggesting that the inhibition of IDO1 might also be of value [33].

A third approach to augment interferon’s anti-tumor effects could involve combining it with other agents capable of inducing immunogenic cell death (ICD). Induction of ICD allows potential tumor antigens to be released and then presented to T-cells by dendritic cells in an immunostimulatory environment [34]. Although we observed that IFNβ was able to induce some tumor cell death (the degree depending on the cell line), this was a rather weak effect and supports previous data suggesting that many tumor cells are resistant to type I interferons’ cytotoxic effects [35]. It is thus possible that co-delivering mRNAs that have been reported to more efficiently cause ICD might increase the local and abscopal anti-tumor effects of IFNβ. For example, data have been published suggesting that ICD can be caused by mRNAs encoding for proteins that induce necroptosis (i.e., MLKL) or pyroptosis (i.e., gasdermins) [36,37], which are potent forms of ICD. Augmentation of the in situ vaccination process could also potentially be improved by inducing more systemic immunostimulatory effects and/or alterations of the TME in non-injected (metastatic) tumors and improvement of abscopal effects.

## 5. Conclusions

In summary, the delivery of IFNβ mRNA LNPs through intratumoral injection has significant anti-tumor activity in two syngeneic mouse tumor models with no obvious toxicity. These effects can likely be further enhanced by combining these LNPs with agents that cause immunogenic cell death and stimulate generalized systemic immunity.

## Data Availability

Data may be made available upon written request to the corresponding author.

## Supplementary Materials

The following supporting information can be downloaded at: https://www.mdpi.com/article/10.3390/vaccines13020178/s1, Figure S1: Leukocyte composition of two syngeneic tumor models—LLC.ova and MOC1. Established tumors were harvested and digested, and flow cytometry was performed. Of the viable cells, the percentage of leukocytes and leukocyte sub-populations is shown.
