# Supplementary material for: In Situ Tumor Vaccination Using Lipid Nanoparticles to Deliver Interferon-β mRNA Cargo
Source: Vaccines (Basel). 2025 Feb 13;13(2):178. doi: 10.3390/vaccines13020178 (PMC11861666; doi:10.3390/vaccines13020178)
Supplement: Supplementary file 1 [file vaccines-13-00178-s001.zip › vaccines-3431123-supplementary.pdf]

Supplementary Materials

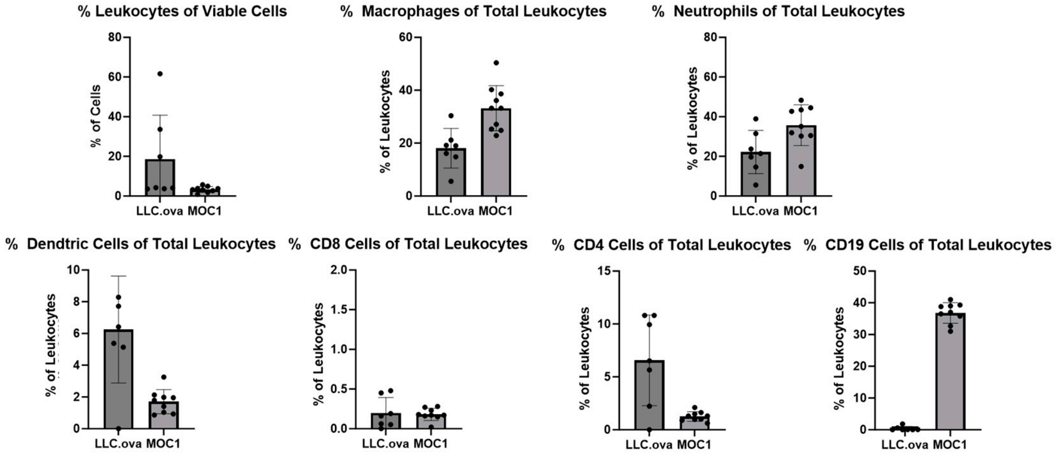

Supplemental Figure 1. Leukocyte composition of two syngeneic tumor models—LLC.ova and MOC1. Established tumors were harvested, digested, and flow cytometry was performed. Of the viable cells, the percentage of Leukocytes and Leukocyte Sub-populations is shown
